# Development of a Graphene-Based Biosensor for Detecting Recombinant Cyanovirin-N

**DOI:** 10.3390/bios10120206

**Published:** 2020-12-16

**Authors:** Pedro Rodrigues de Almeida, André Melro Murad, Luciano Paulino Silva, Elibio Leopoldo Rech, Elmo Salomão Alves

**Affiliations:** 1 Physics Department, Federal University of Minas Gerais, C.P. 702, Belo Horizonte, MG 30123-970, Brazil; elmo@ufmg.br; 2 Federal Center for Technological Education of Minas Gerais, Belo Horizonte, MG 30421-169, Brazil; 3 EMBRAPA Genetic Resources and Biotechnology, Laboratory of Synthetic Biology, Brasília, DF 70770-917, Brazil; andre.murad@embrapa.br (A.M.M.); luciano.paulino@embrapa.br (L.P.S.); elibio.rech@embrapa.br (E.L.R.)

**Keywords:** graphene biosensor, electrochemical sensor, genetically modified soybean, recombinant cyanovirin-N

## Abstract

We present a graphene-based biosensor selective to recombinant cyanovirin-N (rCV-N), an antiviral protein that has proven to be an effective microbicide to inhibit HIV replication. We modified the graphene monolayer devices with 1-pyrenebutanoic acid succinimidyl ester, which interacts with both graphene and the primary and secondary amines of antibodies. By monitoring the change in the electrical resistance of the device, we were able to detect rCV-N in solutions in the range of 0.01 to 10 ng/mL, and found that the detection limit was 0.45 pg/mL, which is much smaller than that obtained with currently available techniques. This is important for applications of this microbicide against HIV, since it may be produced at a large scale from soya bean seeds processed using the available industrial processing technologies. The sensor showed high sensitivity, selectivity, and reproducibility.

## 1. Introduction

The human immunodeficiency virus (HIV) is an infection that attacks the human immune system, and currently is considered by the World Health Organization (WHO) a global epidemic. Data from the WHO indicate that by 2018, there were 37.9 million people worldwide living with HIV, of which 1.7 million were infected only in that year—a 4.5% increase in the number of infected people. To reduce this number, it is important to develop new methods of inhibiting HIV transmission and to improve current methods of prevention. It was shown that cyanovirin-N (CV-N) [1], Griffthsin [2] and Scytovirin [3] are lectins capable of inactivating different strains of HIV, simian immunodeficiency virus and other pathogenic viruses. Analytical studies showed that these molecules are good candidates as additives to topical microbicide gels to prevent the transmission of HIV in macaques [4,5].

Recently, it was demonstrated [6] that recombinant cyanovirin-N (rCV-N), a protein with remarkable stability [7], produced in soya bean seeds has a potent nanomolar anti-HIV activity against HIV-1, which is comparable to the activity range of native CV-N. In addition, soybeans expressing rCV-N can be processed using the available industrial processing technology to produce high-quality feedstock ready to enter the purification process. Thus, a biosensor for this protein will be of great help in an industrial soybean processing for the development of HIV-inhibiting drugs.

Biosensors are widely used for the early stage detection of diseases in humans and plants, and to contribute to the understanding of metabolic processes in these individuals and in microorganisms [8]. Different materials associated with specific mechanisms have been used to develop biosensors [9]. However, there is still an intensive demand to find materials with high selectivity for the development of biosensors with a broader range of applications. Currently, Northern and Western blotting are used for the detection of rCV-N [6]. Blotting is a technique used in molecular biology for the identification of proteins and nucleic acids [10]. All blotting techniques involve a few steps, starting with electrophoresis to separate samples by size. This single step takes a few hours to complete, so it is desirable to have a faster method to detect rCV-N. As far as we know, there is no electrochemical biosensor available for this protein.

Since its discovery in 2004, graphene has emerged as a good candidate as a biosensor transducer [11]. Graphene is a strictly two-dimensional material formed only by carbon atoms arranged in a hexagonal structure [12]. Because of this strictly two-dimensional structure, graphene has a large surface to volume ratio, which enhances the interaction with molecules on its surface and facilitates surface modification to make it selective to different target molecules, a process called functionalization. Many works show that the sensitivity and the detection limit of graphene-based biosensors are equal to or better than those obtained with silicon-nanowires biosensors [13]. With adequate functionalization of graphene, it was possible to produce devices for DNA [14,15,16], detection of cancer molecules [17], Zika virus [18], bacteria and their metabolic activities [19], immunoglobulin aptamers [20], exosomes [21] and *Escherichia coli* [22].

In this work, we report the development of a graphene-based FET biosensor to detect rCV-N in solution. The graphene biosensor showed a high selectivity and sensitivity to rCV-N in solutions with concentrations as low as 10 pg/mL.

## 2. Materials and Methods

### 2.1. Device Fabrication

**Figure 1:**
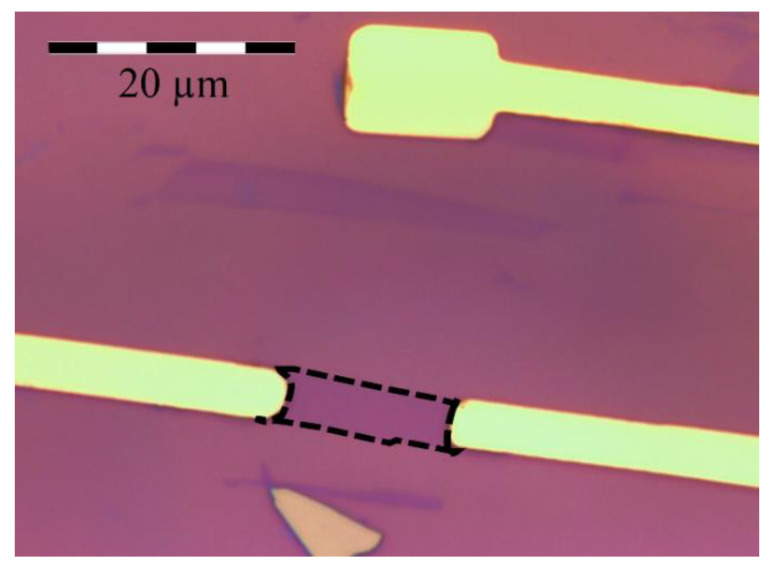
Optical image of a graphene ion-sensitive field-effect transistor (ISFET) showing two electrical contacts (golden areas) to the graphene flake (dashed region) and the gate electrode (top).

We obtained the graphene flakes from natural graphite by the standard micromechanical cleavage technique using an adhesive tape. A graphene device, shown in Figure 1, consists of a graphene monolayer that was transferred onto a heavily doped silicon substrate coated with 300 nm layer of silicon dioxide. We fabricated the ion-sensitive field-effect transistor (ISFET) using conventional microfabrication techniques [23]. Two electrical contacts to the graphene monolayer were formed by thermal evaporation of chromium and gold followed by lift-off. The third electrode on the substrate, near the graphene flake, is used to apply a gate voltage when the device is covered by an electrolyte.

### 2.2. Functionalization

**Figure 2:**
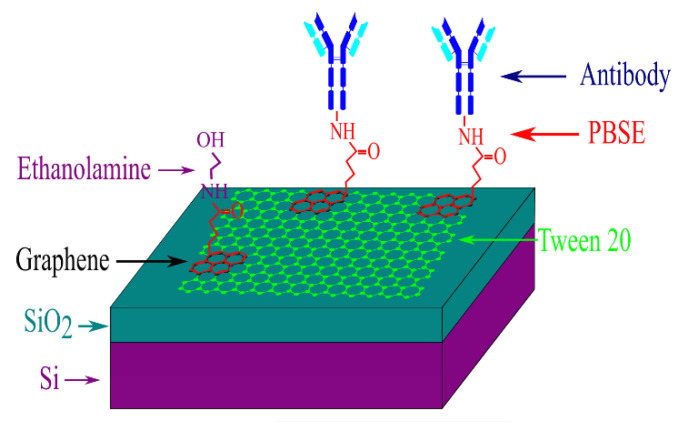
Schematic representation of the device after the functionalization steps described in the text.

To immobilize antibodies onto the graphene monolayer, we incubated the device in a solution of 5 mM 1-pyrenebutanoic acid succinimidyl ester (PBSE) in dimethylformamide (DMF) for 2 h at room temperature [19]. The succinimidyl ester groups are highly reactive with the primary and secondary amines of many proteins, while their pyrene groups bind strongly to graphene via π–π interactions [24,25], as shown schematically in Figure 2.

To achieve conjugation of antibodies to PBSE, we incubated the devices in a solution of 100 µg/mL of antibodies in phosphate buffered saline (PBS), with pH 7.4, at 4 °C, for 20 h. After this, we dipped the devices in ethanolamine for 1 h, at room temperature, in order to deactivate the succinimidyl ester groups not conjugated to antibodies. Finally, we incubated the devices in a 0.1% Tween-20 solution for 1 h at room temperature in order to passivate the uncoated surface of graphene (see Figure 2). After this functionalization procedure, the graphene devices become selective to the target protein due to the “lock and key” complementarity of the antigen-antibody interactions.

**Figure 3:**
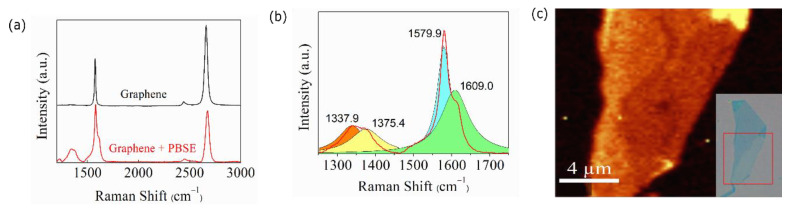
(**a**) Raman spectra of a graphene monolayer before (black curve) and after (red curve) 1-pyrenebutanoic acid succinimidyl ester (PBSE) immobilization. The top curve is shifted for clarity. (**b**) Raman spectrum near G band. (**c**) Raman spectra color map of the intensity of the peak at 1609.0 cm^−1^ for the region of the multilayer graphene flake shown in the inset (red square).

It is desirable that the chemical modification of graphene does not change its band structure to preserve its high sensitivity to proteins after functionalization. To verify the nature of binding between PBSE and graphene we carried out Raman spectroscopy measurements by exciting graphene with a 532 nm laser, before and after treatment with PBSE. The most prominent features in the Raman spectrum of a monolayer of pristine graphene are the *G* and *2D* Raman bands at 1532 and 2716 cm^−1^, respectively [26]. Figure 3a shows the Raman spectra of a graphene monolayer before (black) and after (red) PBSE immobilization. The Raman peak at ~2716 cm^−1^ remains as a single Lorentzian peak after the PBSE treatment, which indicates that the π band and, consequently, graphene’s characteristic electronic properties are not perturbed after functionalization [27]. The shifting of ~15 cm^−1^ of the 2D band to higher frequency is a signature of hole doping by PBSE [28,29].

In the frequency region around the G band, shown in Figure 3b, several peaks related to PBSE appear. The peak at 1337.9 cm^−1^ (orange) is due to sp^3^ bonding, the one at 1375.4 cm^−1^ (yellow) is related to disorder introduced by the hybridization of PBSE with the graphene monolayer, and the peak at 1609 cm^−1^ (green) is attributed to the aromatic pyrene group of PBSE [27,28,29].

Figure 3c shows the spatial Raman spectra color map of a multilayer graphene flake (see inset) after functionalization with PBSE. The map was carried out using a 532 nm excitation laser and was constructed by plotting the intensity of the peak at 1609 cm^−1^ using a filter for this frequency. This map shows that PBSE binds to all graphene mono- and multilayers but not to the substrate.

We performed fluorescence measurements to verify the distribution of antibodies on graphene. First, we labeled the antibodies using fluorescein isothiocyanate (FITC), which is an organic molecule that binds to the amino group of the antibodies. The FITC has excitation and emission wavelengths of approximately 495 and 520 nm, respectively, which gives it a green color. The antibodies were labeled by mixing these three solutions: 2 mg/mL of antibodies in a phosphate buffer (pH = 7.4); a borate buffer solution (pH = 9); and 1 μg/μL of FITC in dimethylsulfoxide. This mixture was incubated at 37 °C, for 90 min [30].

**Figure 4:**
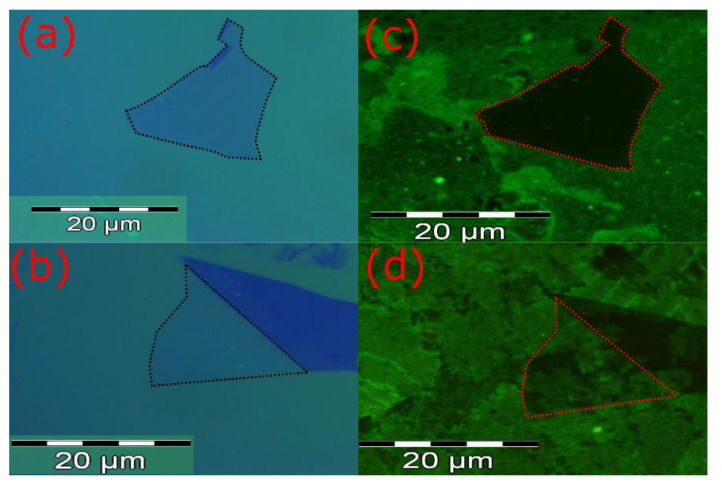
Optical microscope images of graphene flakes (**a**) without PBSE and (**b**) with PBSE, both on top of a SiO_2_/Si substrate. (**c**,**d**) Fluorescence images of the flakes after incubation in a solution of antibodies labeled with fluorescein isothiocyanate (FITC).

Figure 4a,b show optical microscope images of graphene flakes on top of a SiO_2_/Si substrate. The outlines of the flakes are indicated by dashed lines. The darker blue region next to the flake in Figure 4b is a thicker graphite flake which is not important for this work. PBSE was immobilized only on the graphene flake in Figure 4b by following the procedures described in this section. Then, both samples were incubated in the solution of antibodies labeled with FITC.

Figure 4c,d show the results of the fluorescence measurements. No fluorescence was observed from the graphene without PBSE (Figure 4c), whereas in Figure 4d the fluorescence signal is all over the graphene flake, which indicates that PBSE with the FITC-labeled antibodies covers most of the graphene flake. This is also consistent with the Raman color map shown in Figure 3, which also shows that PBSE binds strongly to graphene [27,28,29].

### 2.3. Proteins Preparation

The recombinant proteins cyanovirin (rCV-N), griffithsin (rGRFT) and scytovirin (rSVN) and polyclonal primary antibodies were supplied by the Molecular Targets Laboratory (CCR, NCI, NIH, Bethesda, MD, USA).

## 3. Results and Discussion

### 3.1. Protein Detection

**Figure 5:**
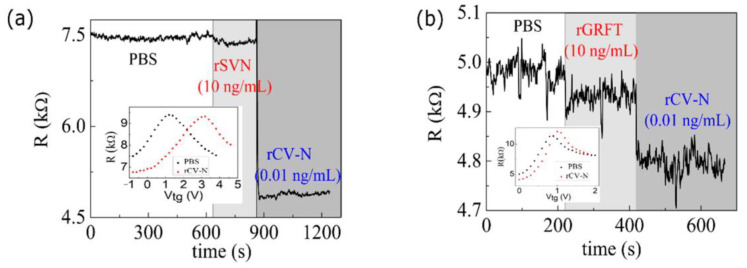
Resistance *versus* time of a graphene ISFET in contact with solutions of (**a**) PBS, rSVN and rCV-N and (**b**) PBS, rGRFT and rCV-N, in this order. The insets show the resistance versus gate voltage plots for the devices covered with PBS (black dots), and, subsequently, with rCV-N (red dots).

We determined the electrical resistance *R* = *V*/*I* of the device by applying an alternating current of amplitude *I* = (100.0 ± 0.1) nA and frequency of (13.3 ± 0.1) Hz, while measuring the voltage amplitude *V* across the device using a lock-in amplifier. A voltage source was used to supply the gate voltage *V*_g_ between the solution and the graphene flake. Figure 5a shows the device resistance versus time as we applied a small droplet of different ionic liquids onto the devices. Starting with a functionalized device, we covered the device with 2 µL of a buffer solution of PBS (pH 7.4). Once the resistance became stable, we removed this solution and applied a solution of 10 ng/mL of rSVN, which had no trace of the target protein, onto the device. The volume applied was enough to cover the graphene and gate regions. As shown in Figure 5a, we observed no significant change in the device resistance between these two steps, even after several minutes. However, when we applied a solution of 0.01 ng/mL of rCV-N onto the device, an abrupt drop in the electrical resistance was observed, which is due to the binding of rCV-N to the functional groups on the graphene surface.

The inset in Figure 5a shows the *R* (*V*_g_) plots measured for the device covered with PBS (black dots), and, subsequently, with rCV-N (red dots). The displacement of the plot to higher gate voltages was due to the additional charge produced by the binding of the target proteins to the antibodies on the surface of the graphene. For this device, the sensitivity is at maximum for *V*_g_ ≈ 0.2 V, where the slope of the *R* (*V*_g_) plot is at maximum. For convenience, the results shown in Figure 5a were measured with the gate grounded, because the drop in resistance for *V*_g_ = 0 V is close to that observed for *V*_g_ = 0.2 V. The interaction between rCV-N and the immobilized antibodies on graphene produces a change in resistance that is ~30 times larger than that produced by rSVN.

We repeated those measurements using the protein rGRFT instead of rSVN for *V*_g_ = 0 V. The results are shown in Figure 5b and the inset shows the plots of *R* (*V*_g_) measured before (black dots) and after (red dots) adding rGRFT to the device. For this device, the best operating point is at *V*_g_ ≈ 0.5 V. However, even when biased at a rather unfavorable condition (*V*_g_ = 0 V), the change in the device resistance was about 4.5 times larger than for rGRFT, despite the additional noise due to this biasing condition. These results indicate that the graphene-ISFET is highly selective to rCV-N. The small drops in resistance observed for rSVN (~0.9 %) and for rGRFT (~1.0 %) are due to graphene regions that were not totally passivated by the solution of Tween-20. Small signals due to nonspecific proteins were also observed for biosensors reported in other works [17,18,20].

### 3.2. Sensitivity of the Graphene Biosensor

**Figure 6:**
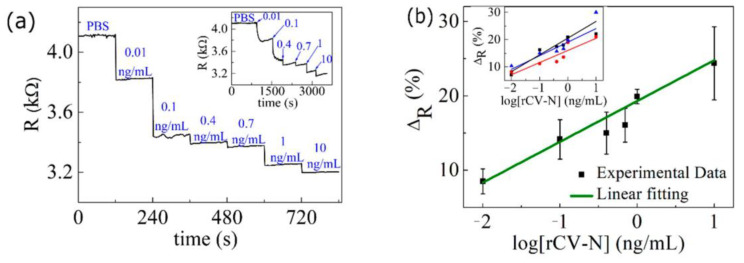
(**a**) Resistance versus time of a graphene-ISFET in contact with solutions with different concentrations of rCV-N. Each plateau corresponds to the last two minutes before a concentration change. The inset shows real time measurement of the electrical resistance for the same concentrations. (**b**) Average of the percentage change in resistance for three devices (shown in the inset) versus log[RCV-N]. The lines are linear fits to the data.

We analyzed the device’s sensitivity by measuring the electrical resistance changes for different concentrations of rCV-N. Initially, we placed a small volume of PBS solution on the device. After the resistance became stable, we applied solutions of rCV-N of different concentrations onto the device at the instants indicated by the arrows in the inset in Figure 6a. After each step, we allowed enough time for the resistance to stabilize. Figure 6a shows the resistance during the two minutes before each change in concentration. We attribute the successive drops in the resistance to the increasing number of bounded protein–antibody sites on graphene as the concentration of rCV-N increases.

We repeated these measurements for three different devices and obtained consistent results. The inset in Figure 6b shows the percentage change ∆_R_ = 100(*R_PBS_* − *R*)/*R_PBS_* in the resistance *R* relative to the resistance *R_PBS_* of each device when covered by PBS solution (blank), as a function of the logarithm of the concentration of rCV-N. Figure 6b shows a plot of the average value of ∆_R_ for the three devices. The error bars are the standard deviation. From a linear regression of these data, we obtained the calibration curve ∆_R_ = (19.3 ± 0.6) + (5.5 ± 0.6) log([rCV-N]), with a correlation coefficient of 0.93 for a detectable range from 0.01 ng/mL to 10 ng/mL. We estimated the detection limit *C*_L_ = 0.45 pg/mL (40.9 fM) for this device by determining the concentration at which ∆*_R_* is equal to the average change in the resistance for PBS solutions (blank) plus 3*σ*, where *σ* = 0.3% is the standard deviation. This detection limit is much lower than that obtained for graphene-based immunosensors for other proteins [17,18,31,32,33] using a similar approximation.

**Figure 7:**
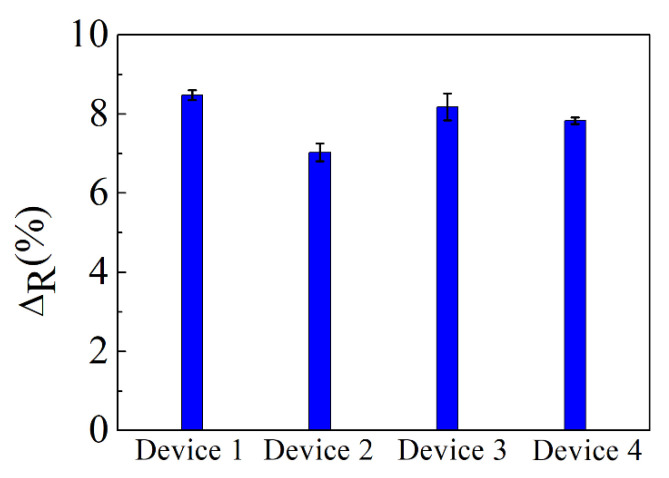
Percentage changes in resistance produced by a solution of 0.01 ng/mL of rCV-N, for four different ISFET devices. The error bars are the standard deviation of the measurements.

To verify the reproducibility of the results, we fabricated another four graphene ISFET devices and placed onto each one a droplet of 0.01 ng/mL of rCV-N. The percentage changes in the resistance of these devices, shown in Figure 7, are consistent with the previous results, with a standard deviation of 7.9%, which indicate the good reproducibility of the sensor.

## 4. Conclusions

We developed a biosensor based on a graphene ISFET with a high selectivity and sensitivity to rCV-N, a protein that is a very promising microbicide against HIV. We have shown that the antibody-rCV-N conjugation changes the electrical resistance of the graphene ISFET with a limit of detection of 0.45 pg/mL (40.9 fM) and a detection range of 0.01 to 10 ng/mL. Due to the relative simplicity of the fabrication process and the high selectivity and sensitivity of the graphene ISFET for rCV-N, this sensor may be used to quantify this protein during the industrial processing of soya bean seeds, which may potentially be used as an anti-HIV resource.
